# Unveiling the nexus: Oncolytic viruses, extracellular vesicles, and immune modulation

**DOI:** 10.1016/j.omton.2025.200940

**Published:** 2025-02-05

**Authors:** Pierre Cordelier

**Affiliations:** 1 Centre de Recherches en Cancérologie de Toulouse, CRCT, Université de Toulouse, INSERM, CNRS, Toulouse, France; 2 Equipe Labellisée Fondation ARC

## Main text

Viruses can easily be considered micromachines, underlining the elegance of nature’s engineering at the nanoscale. Despite their lack of independent life, their ability to parasitize living cells demonstrates unparalleled efficiency, offering both a conceptual framework for understanding their role in biology and a blueprint for harnessing their potential in innovative ways. This designation is particularly fitting for oncolytic viruses (OVs): the increasingly detailed study of their interactions with cancer cells provides invaluable insights into their tropism and lytic activity. This research effort also represents an essential prerequisite for enhancing their utility as programmable molecular tools for patient therapy.

The relationship between viruses and vesicle production is intricate and multi-faceted, involving both the direct manipulation of host cellular pathways and indirect effects on cell biology.^1^^,^^2^ Vesicles, particularly extracellular vesicles (EVs) such as exosomes and microvesicles, play significant roles in viral infection, replication, and pathogenesis. Many viruses manipulate host cellular pathways to enhance the production of EVs. These vesicles can carry viral components (e.g., RNA, DNA, and proteins) or host-derived molecules that facilitate infection or immune evasion. Viral infection also often triggers cellular stress responses, leading to increased vesicle production as part of the cell’s attempt to communicate or manage damage.

Other studies have found that viruses may use vesicles as decoys to distract the immune system, allowing the virus to evade detection and destruction.^3^ Conversely, many studies describe successful partnerships between EVs or exosomes and OVs to improve antitumoral systemic immunity, one of the key determinants of the current appeal of OVs as immunotherapies.^4^^,^^5^ Yet, vesicles as modulators of the host immune response have been associated with both pro-viral and antiviral effects.^6^ On the one hand, viral infection alters vesicle cargo to suppress the immune response. For example, vesicles can carry microRNAs (miRNAs) or proteins that dampen antiviral signaling. Vesicles may also deliver viral proteins to neighboring cells, preparing them for infection or weakening their defenses. On the other hand, host cells may produce vesicles carrying antiviral molecules (e.g., interferon-stimulated genes) as a defense mechanism. Alternatively, vesicles can present viral antigens to the immune system, promoting an adaptive, antiviral immune response.

Examples of the interplay between infection and EVs include HIV, which alters exosome content to suppress immune responses, enabling persistent infection, or SARS-CoV-2, which exploits vesicle pathways to spread viral RNA and modulate immune signaling during COVID-19 infection.^7^ Hepatitis viruses is also known to utilize vesicles to spread infection and evade immune surveillance.^8^ Last but not least, herpesviruses, which resulted in one of the most advanced oncolytic treatments so far, use vesicles to deliver miRNAs that modulate the host cell environment in favor of latent infection.^9^

So far, the interplay between viruses, including those with oncolytic properties, and vesicle production demonstrates the sophisticated ways viruses co-opt cellular pathways to promote their survival and propagation. By understanding these mechanisms, researchers can uncover new therapeutic strategies for antiviral treatments, immune modulation, and cancer therapies using OV.

In line with this, the work of Hirigoyen et al., conducted in Dr. Nicolas Boisgerault’s lab, sheds new light on the interaction between OVs and tumor EVs, particularly their influence on immune system modulation.^10^ First, the authors confirmed that OV infection induces vesicle production, which appear larger than usual, in a range of infected cancer cells from different origins. By doing this, they faced and partially solved one of the key challenges in studying vesicle production during viral infection: eliminating infectious particles from vesicle preparations. This is crucial for gaining a clearer view of the functional consequences of OV-induced EV production. Alternative techniques (e.g., advanced physical separation methods) could further refine these studies.

Using a series of remarkably well-designed experiments, the authors demonstrate that vesicles derived from OV-infected cells carry virally encoded reporter proteins. They speculate that this phenomenon appears to occur spontaneously and may result from the high levels of viral protein production within infected cells. Further co-localization studies could explore whether the biogenesis of vesicles and OVs intersects within infected cells, providing insight into whether this observation is more deterministic than opportunistic.

The authors further discovered that viral infection has a direct impact on EV biology, particularly through viral proteins, such as vesicular stomatitis virus G protein (VSV-G), which integrate into EVs and enhance their internalization by recipient, non-infected cells. Proteomic analyses revealed that these vesicles contain antiviral factors and proteins associated with adaptive immunity (e.g., Melan-A). Notably, these vesicles partially enhanced CD8+ T cell activity *in vitro*. These intriguing and provocative findings highlight the need for further investigation into the immune potential of OV-induced EVs in an *in vivo* context. This would help to fully determine the physiological relevance of these observations and elucidate the functional consequences of oncolytic infection on the antitumoral immune response.

Another compelling observation is the identification of proteins involved in antiviral signaling within OV-induced EVs. This finding suggests a potential role for these vesicles as paracrine mediators of resistance to virotherapy. Testing this hypothesis would require purifying these EVs, treating cancer cells to potentially induce an antiviral state, and subsequently evaluating their susceptibility to OV infection. This functional “duplicity” is reminiscent of the role of cancer-associated fibroblasts (CAFs) in OV infection of cancer cells. On one hand, CAFs have been shown to act as reservoirs for OV replication,^11^ while in other cases, they function as antiviral “sentinels,” restricting viral replication in cancer cells.^12^

Nonetheless, the work by Hirigoyen et al. represents a significant milestone in our understanding of the intricate relationship between OVs and EVs, highlighting the sophistication of viral manipulation of host cellular pathways. By demonstrating the immune-stimulatory potential of OV-induced EVs, this work lays the groundwork for future research aimed at leveraging these interactions to enhance cancer immunotherapies. Moving forward, refining techniques for EV isolation and expanding *in vivo* studies will be critical to fully unravel the therapeutic implications of these findings and design more precise and effective OV-based treatments.

## Acknowledgments

The author is thankful for financial support from 10.13039/501100004097Fondation ARC, Programme Equipes labellisées Fondation ARC (2024-2026).

## Declaration of interests

The author wishes to confirm that there are no known conflicts of interest associated with this publication and that there has been no significant financial support for this work that could have influenced its outcome.

## Declaration of generative AI and AI-assisted technologies in the writing process

During the preparation of this work, the author used ChatGPT in order to improve language and readability. After using this tool/service, the author reviewed and edited the content as needed and takes full responsibility for the content of the publication.
